# Oral rabies vaccination of dogs—Experiences from a field trial in Namibia

**DOI:** 10.1371/journal.pntd.0010422

**Published:** 2022-08-22

**Authors:** Conrad Martin Freuling, Frank Busch, Adriaan Vos, Steffen Ortmann, Frederic Lohr, Nehemia Hedimbi, Josephat Peter, Herman Adimba Nelson, Kenneth Shoombe, Albertina Shilongo, Brighton Gorejena, Lukas Kaholongo, Siegfried Khaiseb, Jolandie van der Westhuizen, Klaas Dietze, Goi Geurtse, Thomas Müller

**Affiliations:** 1 Institute of Molecular Virology and Cell Biology, Friedrich-Loeffler-Institut (FLI), WHO Collaborating Centre for Rabies Surveillance and Research, WOAH Reference Laboratory for Rabies, Greifswald-Insel Riems, Germany; 2 Institute of International Animal Health/One Health, Friedrich-Loeffler-Institut (FLI), Greifswald-Insel Riems, Germany; 3 Ceva Innovation Center GmbH, Dessau–Rosslau, Germany; 4 Mission Rabies, Cranborne, United Kingdom; 5 Animal Disease Control, Kunene, Ministry of Agriculture, Water & Land Reform, Directorate of Veterinary Services, State Veterinary Office, Opuwo, Namibia; 6 Directorate of Veterinary Services, State Veterinary Office, Omusati, Namibia; 7 Directorate of Veterinary Services, State Veterinary Office, Ondangwa, Namibia; 8 Deputy Chief Veterinary Officer, Animal Disease Control, North, Ministry of Agriculture, Water & Land Reform, Directorate of Veterinary Services, State Veterinary Office, Ongwediva, Namibia; 9 Chief Veterinary Officer, Directorate of Veterinary Services, Ministry of Agriculture, Water and Land Reform, Windhoek, Namibia; 10 Faculty of Agriculture and Natural Resources, Ogongo Campus, University of Namibia, Wnindhoek, Namibia; 11 Central Veterinary Laboratory, Directorate of Veterinary Services (DVS), Ministry of Agriculture Water and Land Reform, Windhoek, Namibia; 12 SWAVET, Windhoek, Namibia; Animal Health and Veterinary Laboratories Agency: Animal and Plant Health Agency, UNITED KINGDOM

## Abstract

Dog-mediated rabies is responsible for tens of thousands of human deaths annually, and in resource-constrained settings, vaccinating dogs to control the disease at source remains challenging. Currently, rabies elimination efforts rely on mass dog vaccination by the parenteral route. To increase the herd immunity, free-roaming and stray dogs need to be specifically addressed in the vaccination campaigns, with oral rabies vaccination (ORV) of dogs being a possible solution. Using a third-generation vaccine and a standardized egg-flavoured bait, bait uptake and vaccination was assessed under field conditions in Namibia. During this trial, both veterinary staff as well as dog owners expressed their appreciation to this approach of vaccination. Of 1,115 dogs offered a bait, 90% (n = 1,006, 95%CI:91–94) consumed the bait and 72.9% (n = 813, 95%CI:70.2–75.4) of dogs were assessed as being vaccinated by direct observation, while for 11.7% (n = 130, 95%CI:9.9–17.7) the status was recorded as “unkown” and 15.4% (n = 172, 95%CI: 13.4–17.7) were considered as being not vaccinated. Smaller dogs and dogs offered a bait with multiple other dogs had significantly higher vaccination rates, while other factors, e.g. sex, confinement status and time had no influence. The favorable results of this first large-scale field trial further support the strategic integration of ORV into dog rabies control programmes. Given the acceptance of the egg-flavored bait under various settings worldwide, ORV of dogs could become a game-changer in countries, where control strategies using parenteral vaccination alone failed to reach sufficient vaccination coverage in the dog population.

## Author summary

Rabies in dogs can be prevented by vaccination, and this approach has become a cornerstone in the control and eventual elimination of the disease. However, vaccinating hard-to-reach often free-roaming dogs are a challenge and represents one of the challenges to reach sufficient herd-immunity. A potential solution would be to vaccinate these dogs using oral baits filled with a vaccine. In this study we have assessed the acceptability of oral rabies vaccination (ORV) in Namibian dogs under field conditions. The results demonstrate that the method is acceptable both for the owners and the dogs, with a very high uptake of the egg-flavored bait. This supports the potential of ORV to contribute to vaccination programs where parenteral vaccination alone failed to reach sufficient vaccination coverage in the dog population.

## 1. Introduction

The Tripartite (WHO, OIE and FAO) considers rabies control a priority but also an entry point to strengthen the underlying systems for coordinated, collaborative, multidisciplinary and cross-sectoral approaches to the control of health risks at the human-animal interface [1]. Among the various mesocarnivorous and chiropteran rabies reservoir hosts [2,3], domestic dogs pose by far the greatest threat to global public health [4,5]. Mass dog vaccinations and public awareness are key to success. Vaccinating at least 70% of the targeted dog population would break the cycle of transmission within the dog population and from dogs to humans saving the lives of several tens of thousands of people [6]. While concerted control measures at national and supranational levels have been successful at eliminating dog-mediated rabies in upper-income countries in Europe and North America [7,8], over the past three decades Latin America and the Caribbean have made impressive progress in controlling the disease at the animal source [9,10]. In 2019, Mexico was the first country to declare freedom from dog-mediated rabies [11], while the remaining countries in this region are on the cusp of eliminating rabies deaths or even in the endgame of dog rabies elimination [12]. Despite these successes, dog-mediated rabies continues unabated in Africa and Asia and is responsible for an estimated 59,000 human deaths annually (95% CI 25,000–159,000) [13]. At present, parenteral vaccination is considered the only approach for addressing dog-mediated rabies at-scale, however, implementing these techniques in resource-poor settings can be challenging. There are increasing reports of the inadequacies of this approach among important subpopulations of susceptible dogs. Perhaps the greatest challenge is maintaining adequate herd immunity in free-roaming dog populations [14–16]. A promising alternative solution to this problem maybe oral rabies vaccination (ORV) [16,17].

For example, ORV has been successfully used in eliminating rabies in wildlife populations. Over the past 4 decades, due to large-scale ORV programs fox-mediated rabies has virtually disappeared in large regions of western and central Europe and Canada [18–20]. Using the same approach rabies epizootics in coyotes and gray foxes in the US could be brought under control [21]. While ORV has been a cornerstone in rabies virus elimination from wildlife populations, oral vaccines have never been effectively used in dog rabies control programs and are still an undervalued tool for achieving dog rabies elimination [16,17]. Although the WHO issued recommendations on ORV of dogs [22], the number of studies is still limited. A few oral rabies vaccine strains have been investigated for ORV in dogs under experimental or confined conditions [23–29].

Attractiveness and uptake of different baits developed for dogs have been tested before [30–39]. While immunogenicity studies in local dogs using different vaccine bait combinations have been conducted in among others Tunisia [40,41], Turkey [42], India [43], Namibia [44] and Thailand [45], at least one efficacy study met international standards applicable at that time [43]. However, only few field applications have been documented so far [42,46–50].

With the launching of the Global Strategic Plan for elimination of dog-mediated human rabies deaths by 2030 [51], the concept of ORV in dogs gained momentum again to be employed as a complementary approach to current, traditional mass dog vaccination efforts [52].This strategy is currently promoted by the WHO and the OIE [16], but with the exception of Thailand [50], field data on its applicability and effectiveness under various socio-economic settings are lacking. Presently, a dog rabies elimination program using mass vaccination campaigns is implemented in the Northern Communal Areas (NCAs) of Namibia where the percentage of owned but free-roaming dogs is high [53]. Also, follow-up investigations indicated that the vaccination coverage reached was below the thresholds needed for rabies control and elimination [54]. Therefore, we set out to implement an ORV pilot field study using a 3^rd^ generation oral rabies vaccine with a high safety profile according to international standards to demonstrate the applicability of this approach in Namibia, potentially serving as a blueprint for other regions in Africa, and beyond, where dog rabies is still endemic and the accessibility of the target population is a key constraint. The objectives of this study were to test the feasibility and benefits of ORV in dogs as a potential complementary tool within the rabies programme in Namibia by assessing bait uptake and vaccination rate in Namibian dogs and the acceptance of the method by veterinary authorities and local dog owners.

## 2. Materials and methods

### 2.1. Ethics statement

The implementation of the ORV field trial was an integral part of the official national canine rabies control program under leadership of the Namibian Directorate of Veterinary Services (DVS) in the Ministry of Agriculture, Water, Forestry and Land Reform (MAWLR) [53,55]. Approval to use the non-licensed vaccine in the frame of a disease control trial was granted by the Chief Veterinary Officer of the DVS at the MAWLR, Namibia. Data from an immunogenicity study showing non-inferiority of the immune response after oral vaccination to parenteral vaccines in local Namibian dogs [44], a human risk assessment for the specific live-attenuated vaccine virus [56] and the submission of a detailed study plan to DVS were basic prerequisites for decision taking. Importation of the oral rabies vaccine was authorized by the Namibian Medicine Regulatory Council (NMRC) under section 31(5) (c) of the Medicines and Related Substances Control Act 2003—registration number: 17.12.20/PW/2021/IMPORT-L/0009/ek. Under this permission, the vaccine baits were imported via SWAVET Namibia.

Additional approval from the Namibian ethics committee was not required since no personal data of dog owners were obtained. Approval of the field trial by DVS was given under the premise that the purpose of this pilot field trial had to be explained to dog owners and that the dog owner had previously given his/her formal verbal consent that his/her dog(s) could be offered a vaccine bait. To this end, dog owners were given a specific leaflet with ORV related information provided in both the official (English) as well as the local (Oshiwambo) language and issued a certificate of bait consumption that also contained an emergency contact phone number in case of any adverse events.

### 2.2. Study sites

**Fig 1:**
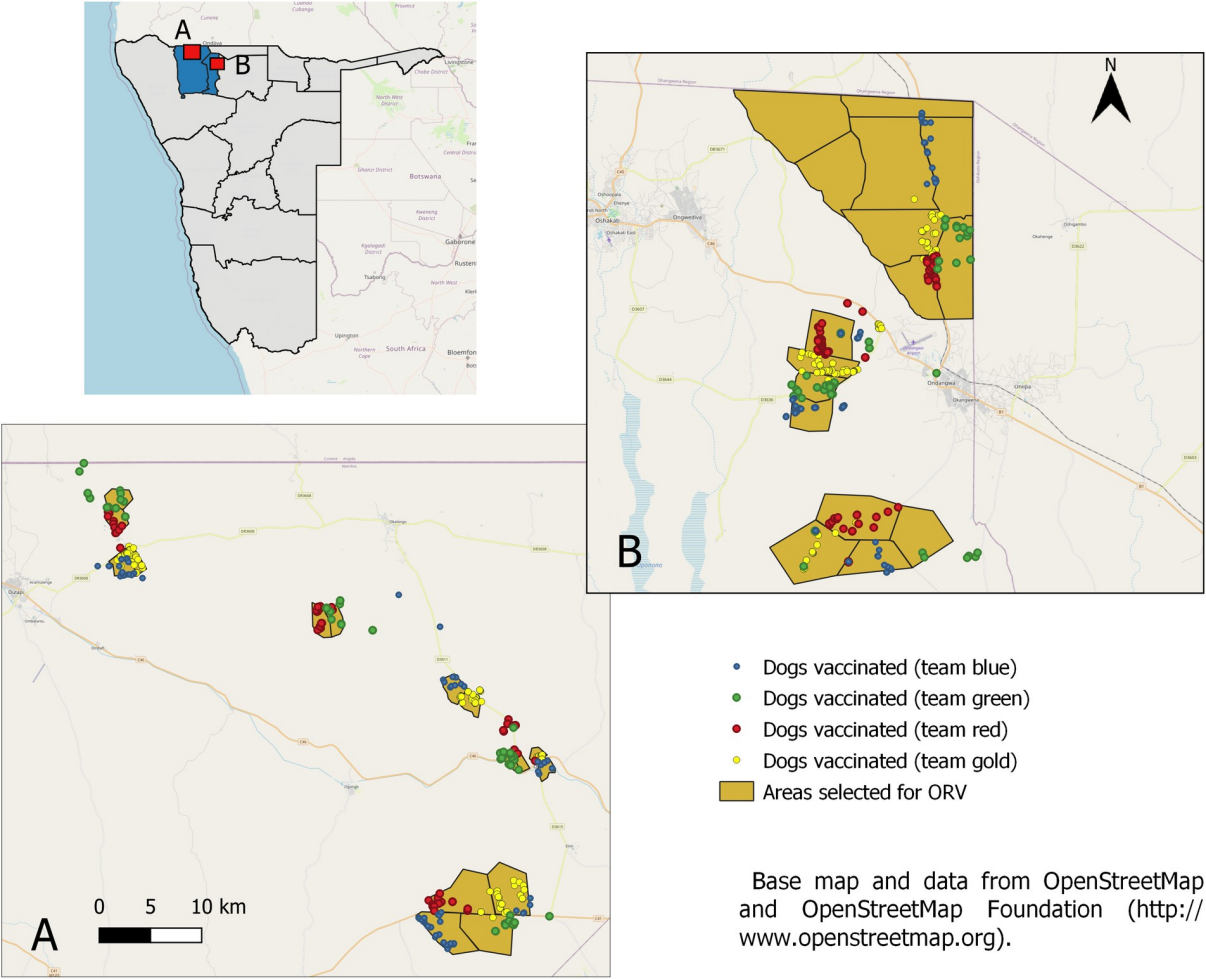
Map of Namibia (left) and the area of the field trial enlarged (right) for Omusati (A) and Oshana (B), with color-codes used for the individual teams. This map contains information from OpenStreetMap and OpenStreetMap Foundation (https://www.openstreetmap.org/#map=6/-23.544/17.842) which is made available under the Open Database License (https://www.openstreetmap.org/copyright).

The ORV field trial was conducted in the NCAs, in different rural and suburban communities within the Omusati and Oshana regions (Fig 1). While in Omusati, more than 90% of the population live in rural areas, ppredominately living from subsistence crop and livestock farming, in Oshana, the economic centre of the north, 45% live in urban areas, and only for 13% of the population farming is the main source of income. The average population density in Omusati and Oshana is 9.39 people/km^2^ and 35.77 people/km^2^, respectively [57].

The field trial areas were selected after consultation with the Directorate of Veterinary Services (DVS) considering available infrastructure and logistics (Oshana—headquarters) and based on results of a Knowledge, Attitude and Practice (KAP) study conducted, indicating low vaccination coverage in certain regions due to free-roaming hard-to-reach shepherd dogs. These dogs accompany the movement of cattle herds, partly even across the border to Angola, and are often difficult to handle by their owners and vaccination teams.

### 2.3. Vaccine baits

Oral rabies vaccinations were conducted using 3^rd^ generation oral rabies virus vaccine (Ceva Innovation Center GmbH, Dessau in Germany) consisting the SPBN GASGAS vaccine virus strain, a genetically engineered derivate of SAD L16 derived from the vaccine strain SAD B19 which is licensed for foxes and raccoon dogs according to international standards (Freuling et al., 2019). The recombinant vaccine virus construct is distinguished from SAD B19 by the deletion of pseudogene ψ, the introduction of four recognition sequences for restriction enzymes and duplicate insertion of an identical altered glycoprotein [58]. The genes encoding for glycoprotein G contain the amino acid exchange Arg333→Glu333 and Asn194→Ser194 to eliminate residual pathogenicity and reduce the risks for compensatory mutations, respectively [59]. These alterations, significantly enhance the safety profile of the vaccine virus [60]. A soft sachet filled with the liquid vaccine virus (3 mL, 10^8.2^ FFU/mL) was incorporated in a universal industrial manufactured egg-flavored bait (egg bait) previously shown to be highly attractive to local free-roaming dogs [38,39,61,62]. Immunogenicity of the vaccine baits had been demonstrated in local Haitian, Thai and Namibian dogs before [44,45,48].

Based on field experience, acceptance of the egg bait was further optimized by dipping them into locally available commercial tuna- or chicken liver-flavored cat liquid snacks immediately before offering the bait to the dog [50].

### 2.4. Shipment, transportation and storage of vaccine baits

Vaccine baits were shipped according to IATA guidelines on dry ice (UN 1845) directly from the manufacturer to the Central Veterinary Laboratory (CVL), Windhoek, using a commercial courier service. After temporary storage at CVL the vaccine baits were further transported to the Ondangwa branch of CVL located in the Oshana region of the NCAs. Upon arrival in Windhoek and at the field study areas the vaccine baits were stored in standard style freezers at -18 to -20°C until further transportation or use in the field, respectively. Maintenance of the cold chain was ensured and documented using temperature data logger and integrated electronic measuring. Prior to shipment and the prior to start of the field trial, the quality of the baits and the vaccine titre was checked independently by the national and OIE reference laboratory at the Friedrich-Loeffler-Institute (FLI) essentially as described [63].

### 2.5. Vaccination teams

Immediately prior to the field trial, a two day staff introduction session and workshop was conducted during which staff was trained on the objectives of the field trial, oral rabies vaccination, vaccine bait handling, safety issues, techniques for approaching free-roaming dogs, best practice on offering vaccine baits to dogs, electronic data collection (bait handling by individual dogs—duration, consumption, perforation and/or swallowing of sachet), and interpreting effectiveness of vaccination attempt. The importance of retrieving the discarded vaccine sachet after bait consumption as described [50] was highlighted followed by a door-to-door vaccination training in the field.

There were four vaccination teams working simultaneously, with each team consisting of two DVS staff members (state veterinary officer, animal health technician), a data collector and an international expert. While the state veterinary officers were responsible for contacting dog owners, explaining the purpose of the study, seeking owners consent and issuing a certificate of bait consumption, the animal health technicians acted as vaccinators. Data collectors comprised faculty members and students from the Faculty of Agriculture and Natural Resources, Ogongo Campus, University of Namibia (UNAM). Vaccination teams used four-wheel drive pick-up trucks equipped with coolboxes, cooling bags, gloves, rubbish bags, and disinfectants.

### 2.6. Vaccinations

Vaccination campaigns were announced via local radio the evening before and the morning the campaigns took place. Both door-to-door as well as central-point vaccinations were conducted. Vaccine baits were distributed to the targeted dog population using the hand-out and retrieve model [61]. The field trial was carried out at the end of the dry season during the second half of October 2021. During this time, vaccinations were performed over eight full working days (including two half days).

Vaccine baits were transferred to portable cool boxes the evening before field use, allowing them to thaw before they were offered to the dogs. Baits unused at the end of the vaccination day were kept at refrigerator temperatures (4–8°C) and offered to dogs the next day to avoid repeated freezing and thawing of vaccine baits. Baiting was conducted both at individual homesteads as well as at central places in villages where people brought their dogs for oral rabies vaccination. Vaccination took place between 8:00 am and 6:00 pm. Team debriefings and daily evaluations were held at the end of each vaccination day.

Vaccination team members handing the baits (vaccinators) wore examination gloves. Dog owners were informed that dogs offered a bait should be left alone for 12 hours to minimize potential contact with the live vaccine virus. Any discarded sachet was retrieved, collected in trash bags and disposed of as infective materials at the Ondangwa branch of the CVL according to prevailing regulations on hazardous waste.

### 2.7. Data collection and vaccination monitoring

For collection of vaccination and survey data as well as project management, e.g. navigation within demarcated boundaries, sharing real-time team locations during roaming work and survey assessment, a smartphone application including the web-based backend platform was used essentially as described [64]. The App was provided by Mission Rabies, a non-governmental organization specializing in large scale rabies control (https://missionrabies.com/). Smartphones with WVS version of the Mission Rabies App installed were provided to each team. Survey related data including dates, owner consent, size (small = <10kg; medium = 10-30kg; large = >30kg), sex and number of dogs per household, dogs vaccinated and bait handling by individual dogs, i.e. duration, consumption, perforation and/or swallowing of sachet, and the resulting assumed vaccination status (vaccinated, non-vaccinated, unknown) were recorded on the phones using questionnaire forms, pre-designed by an administrator on the backend platform and remotely loaded to the handsets using 3G. Data were entered offline and stored locally on the handset where it could be reviewed on a map the same day. The app was also used to assign working zones for each vaccination team (different colours–gold, red, green and blue) on the App backend platform the day before with demarcated boundaries for each zone automatically synchronized to the App on each teams’ handset via internet connection.

### 2.8. Evaluations and statistical analysis

A dog was considered ‘interested’ if the animal had any direct contact (smelling, licking) with the bait offered, irrespective of subsequent handling. Animals were regarded as successfully ‘vaccinated’ if the bait chewing and intensity (thoroughness) was detectable and/or perforation of the sachet clearly visible. Any dog that swallowed the bait immediately, or walked away with it and could not be observed, or chewed inappropriately on the bait without visible perforation of the sachet was assigned an ‘unknown’ vaccination status. The status ‘non-vaccinated’ was assigned if the dog was not interested or the bait was only shortly taken up and immediately dropped with the bait casing and sachet still intact. The latter also applied to dogs that showed interest (and accepted the bait) but were interrupted by external factors (other dogs, humans, cars, etc) and discontinued bait handling. The maximum observation time for a dog was three minutes.

Data were uploaded daily to a cloud-based server and downloaded by evaluation supervisors as an Excel document Microsoft Excel 2013 (Microsoft Corporation, Redmond, WA, USA) for initial review and analysis. Spatial information was analyzed and displayed using QGIS Geographic Information System (QGIS.org, 2022.http://www.qgis.org) with base map and data from OpenStreetMap and OpenStreetMap Foundation (http://www.openstreetmap.org).

Statistical analysis was performed first by univariate contingency table testing (Chi^2^—and Fisher’s exact test) and followed by a multiple logistic regression (MLR). The dependent variable was “vaccination success” (yes/no), and datasets for dogs with an “unknown” status had to be removed. Independent variables were date, period of the day, team, level of supervision, if the dog was alone or together with other dogs, size and sex of dogs. Variables with p ≤ 0.20 (univariate analysis) were included into the final MLR model. This cut-off value (p = 0.20) instead of the standard 0.05 was selected for the univariate analysis as recommended [65], the latter can fail in identifying variables known to be important in a multivariable analysis. Statistical analyses were carried out using GraphPad Prism v9.0 (GraphPad Prism Software Inc., San Diego, USA).

## 3. Results

An exceptionally high percentage of dog owners (99%) agreed to have their dogs vaccinated with this novel technique and vaccine bait. Of ten households contacted where vaccination was not conducted because of missing consent, in seven cases the owner was absent, in one case there was no person above 18 years of age available and two dog owners refused to get their dog vaccinated. Using the mobile planning and data capturing technology, a total of 1,139 datasets were generated.

The majority of dogs (78%) encountered and offered a bait during the study were owned and free-roaming. The proportion of ownerless free-roaming dogs was 3%, while the remaining dogs were assessed as confined during the vaccination. With 63%, there was a gender bias towards male dogs. Larger dogs (>30 kg) were rare (8%), whereas medium (57%) and small (<10kg; 33%) were dominating in the dog population. The majority of dogs (80%) were offered baits at the individual homesteads while the others were baited at central places (crush-pens, village centres, etc.) in respective areas (Fig 1).

**Fig 2:**
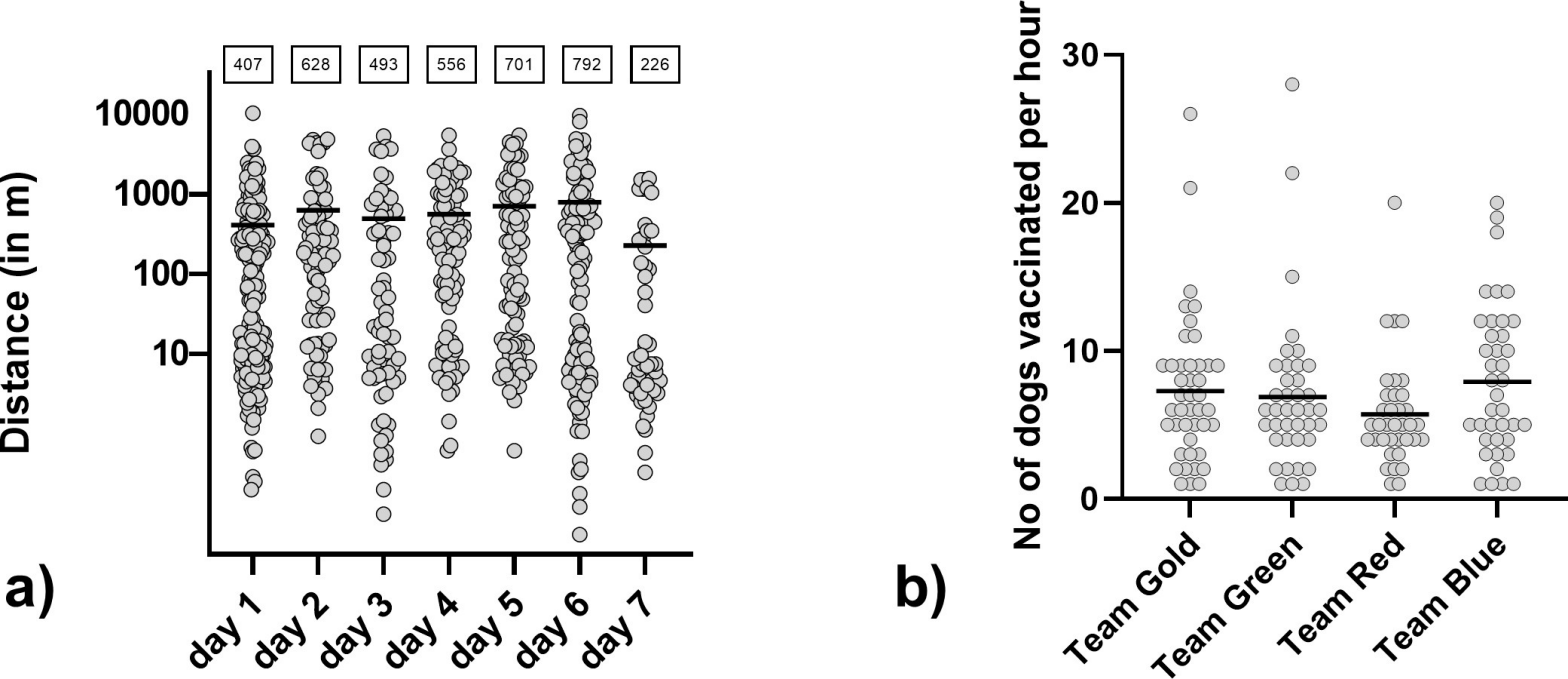
Euclidian distance between consecutively baited dogs per day as calculated by their individual GPS-tracked position (a), with the mean indicated (value shown in boxes). Number of dogs vaccinated per hour and team (b).

The mean distance between individual baitings per team was 533m, with the lowest mean distance (226m) at the last day of the study when semi-urban areas were included. The longest distance between two baitings was 10km (Fig 2A). Overall, under field study conditions, the average number of dogs vaccinated per hour was 7, with a maximum of 28 dogs vaccinated per hour for one team (Fig 2B).

**Fig 3:**
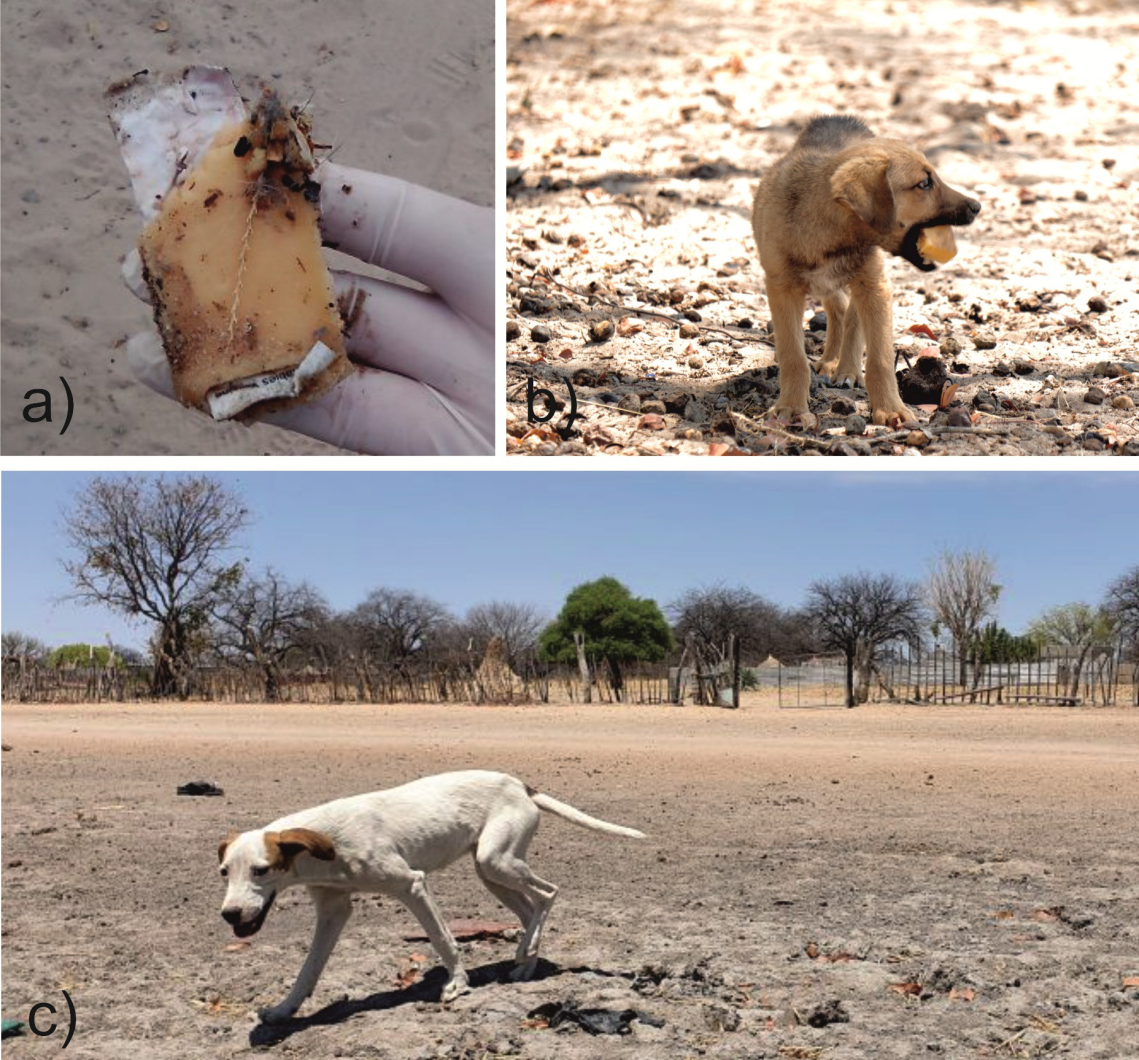
Remains of a partially consumed bait with the blister perforated (a). A puppy consuming a bait (b). Visual impression from a traditional homestead where dogs were vaccinated (c).

Of 1,115 dogs offered a bait, 93.6% (n = 1,044, 95%CI:92.0–94.9) were interested and 90% (n = 1,006, 95%CI:91–94) consumed the bait (Fig 3). Overall, 72.9% (n = 813, 95%CI:70.2–75.4) of dogs were assessed as being vaccinated, for 11.7% (n = 130, 95%CI:9.9–17.7) the status was recorded as “unknown” and 15.4% (n = 172, 95%CI: 13.4–17.7) were considered as being not vaccinated. In 54.9% (n = 552) of dogs observed, the vaccine blister was swallowed, while 43.4% (n = 437) of dogs that consumed a vaccine bait discarded the blister. For the remaining dogs, the status of the bait could not be verified, as e.g. the dog ran away with the bait and could not be observed anymore. Only 9.8% (N = 43) of all blisters retrieved were not perforated.

**Fig 4:**
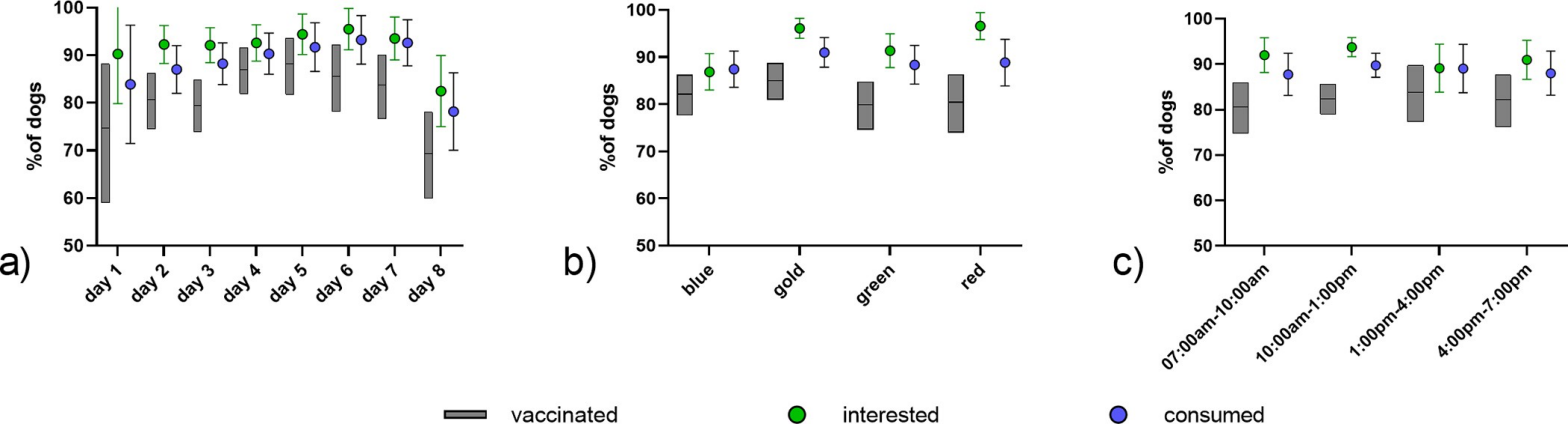
Comparison of bait interest, bait consumption and vaccination per study day (a), daytime (b), and team (c). The mean and the 95% confidence limits are indicated.

For the statistical analysis, 985 entries with a vaccination assessment (yes/no) were available. A statistically lower vaccination rate (p = 0.0048, Chi-square test) was observed on the last (69.8%) and first day (76.7%) of the campaign (Fig 4A). Differences in vaccination rates during time of the day (Fig 4B) and the different teams were not significant (Fig 4A).

**Fig 5:**
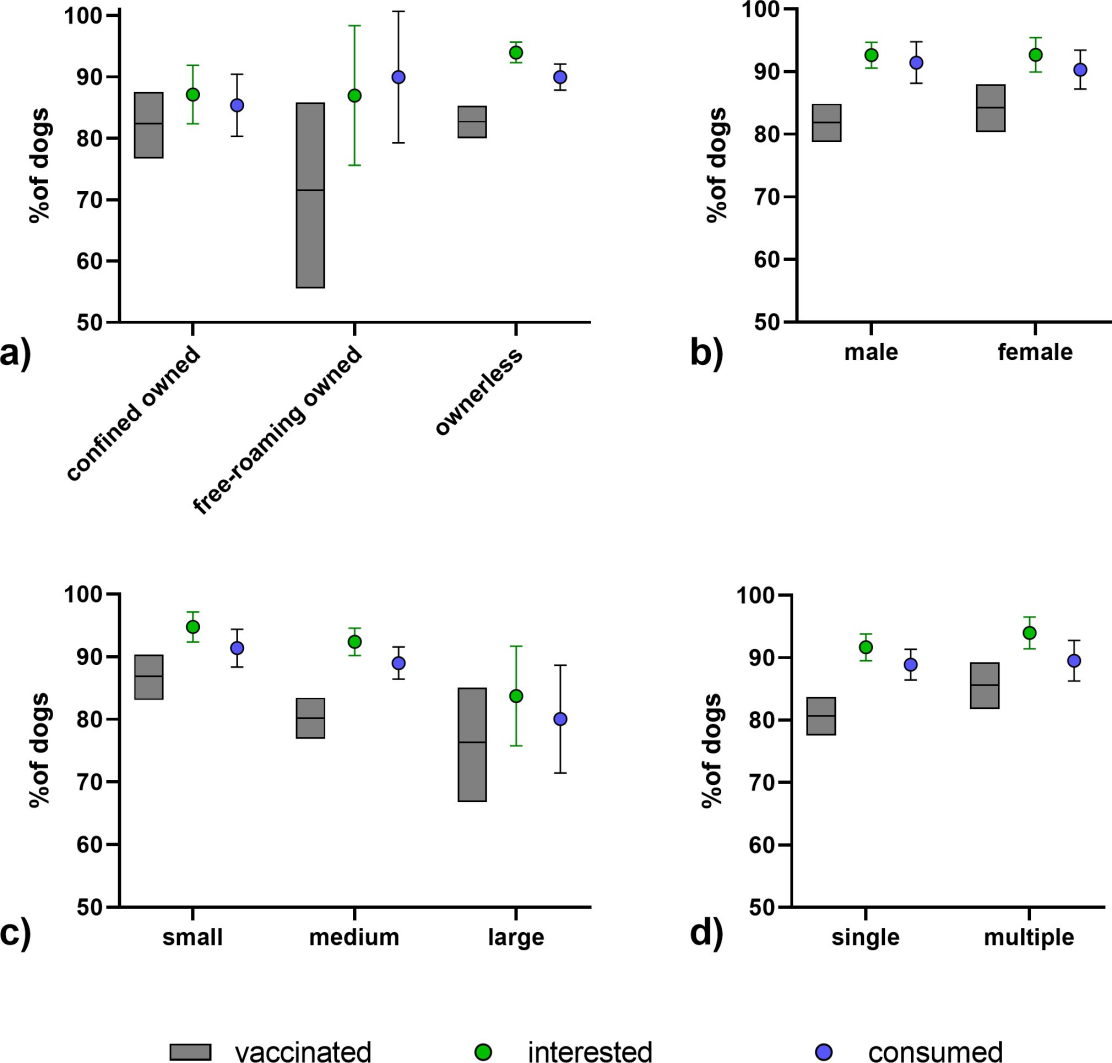
Comparison of bait interest, bait consumption and vaccination according to dog owner status (a), sex (b), size of the dog (c), and the social setting (d). The mean and the 95% confidence limits are indicated.

While there was no statistical difference in vaccination status in regard to the confinement status (Fig 5B and S1 Table) or the sex of the dog (Fig 5B), smaller dogs (p = 0.0166, Chi-square test) and dogs offered a bait with multiple other dogs being present (p = 0.0494, Fisher’s exact test) had significantly higher vaccination rates (Fig 5C and 5D). All variables with a p<0.20 identified, i.e. date, size and social situation of the dog, in the univariate analyses were included in a multivariate logistic regression model, but only size and social situation had a significant impact (S2 Table). Vaccination success was higher in small dogs and when more than one dog was present and was offered a bait.

**Fig 6:**
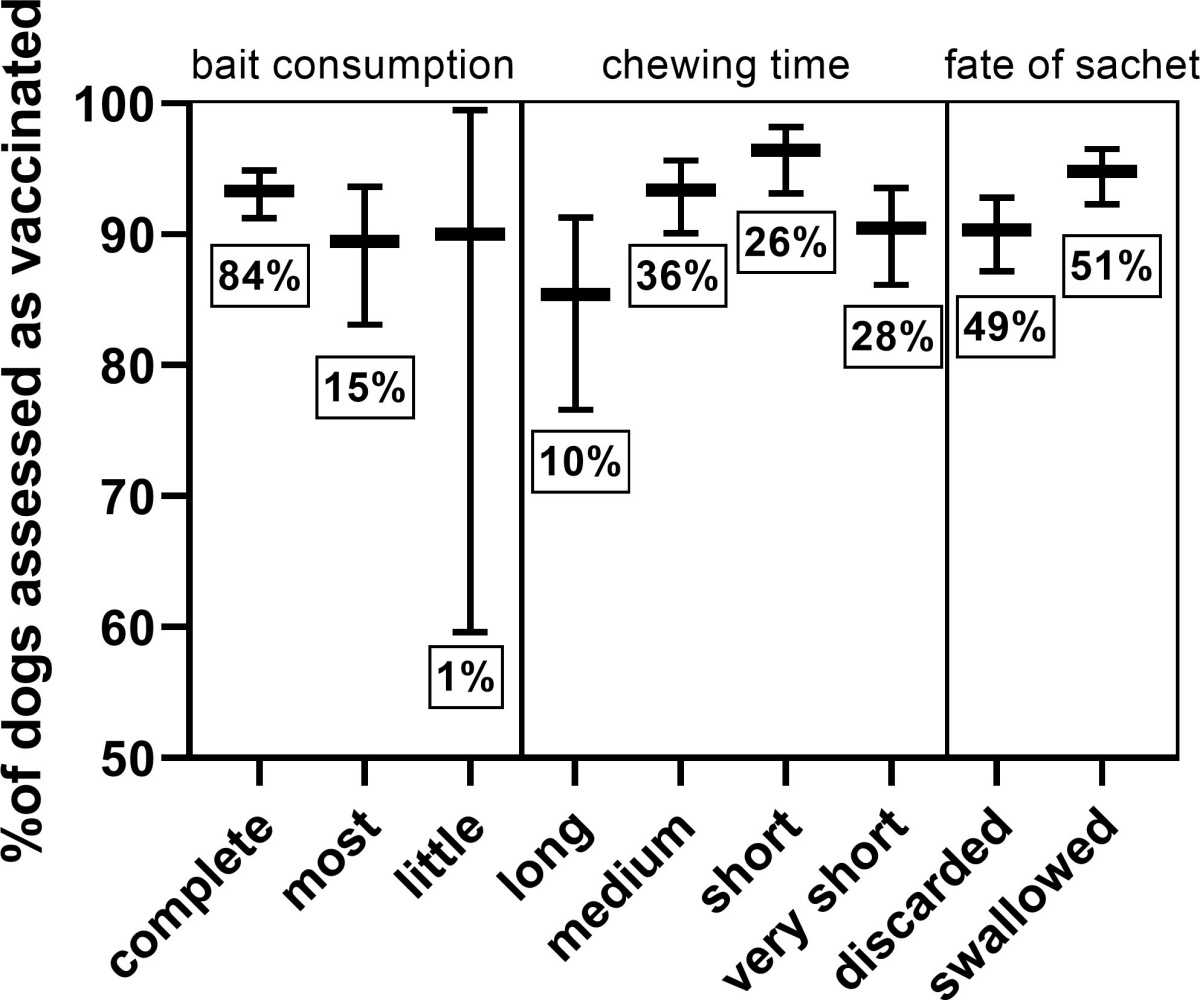
Comparison of vaccination success according to bait consumption, chewing time, and the fate of the sachet. The mean and the 95% confidence limits are indicated. The percentage of total dogs per assessment is given below each graph.

The amount of bait matrix consumed did not affect vaccination success. However, the chewing time and fate of the sachet (discarded or swallowed) had a significant effect on vaccination success (Fig 6 and S3 Table). Dogs that chewed long (>60sec) and dogs that discarded the sachet were less likely to be considered vaccinated. Dogs chewing long rarely swallowed the sachet (12.4%), meanwhile most dogs that chewed for a very short time swallowed the sachet (74.2%).

## 4. Discussion

Overall, the results from this first ORV field trial in Namibia demonstrate a high acceptance for this method both by the veterinary/technical staff as well as the dog owners. In the field, the apparent efficiency in vaccinating dogs, particularly those that cannot be easily handled, was well acknowledged both by the veterinary staff involved as well as by the owners of dogs. For many dogs, this was the first time they had ever been vaccinated. Only very few individuals did not give their consent to vaccinate their dog using a novel vaccination approach and a vaccine that is not yet licensed. This is surprising and very promising for future vaccination campaigns in dogs, as for human diseases there seems to be an increasing hesitancy for vaccination, e.g. for COVID-19 [66]. Public announcement prior to the campaign by radio, and the direct interaction with the dog owner by DVS likely played an important role in the acceptance of this approach.

The egg-flavoured baits, additionally dipped with meat-based flavor, were highly attractive to the dogs. This adds to the results of numerous studies, showing a high acceptance rate of ORV baits in dogs, e.g. Navajo Nations, US (77.4%) [38], Goa State, India (77.5%) [33], and Thailand (78.8%) [39] and Bangladesh (84%) [62]. The percentage of dogs offered a bait that were considered vaccinated by ORV in this field trial was at least 72.9% but likely higher, because a number of dogs disappeared with the bait and were considered “unknown”. While about half of the vaccine blisters were swallowed, when blisters were retrieved, more than 90% were perforated, suggesting that if the bait was consumed, a large proportion of dogs have likely had contact with the vaccine and can be regarded as vaccinated. In any case, the observed vaccination rate of dogs offered a bait was slightly lower than with the same bait in Thailand with 83% [39].

Although the assessment of vaccination was based on individual observation, the small differences between teams suggest that the overall bias was not affecting the outcome of the analysis (Fig 2A). Even though temperatures reached 35°-39°C during the early afternoon, this did not affect bait interest, consumption and vaccination success. ORV protocols for dogs in Namibia and likely in other areas with a similar situation can therefor disregard the time of campaign and focus on other parameters to increase effectiveness.

The fact that smaller dogs had a higher interest, consumed baits more readily and showed a higher vaccination rate as opposed to mid-sized and large dogs is interesting. Partly, these small dogs comprised of younger puppies (Fig 3).

Also, in situations when more dogs were around, smaller dogs tended to be more competitive towards consuming the bait, even though several baits were offered to avoid hierarchic feeding behavior. A similar observation was made in Thailand, where small and young dogs had higher bait acceptance rates [39]. Dogs that chewed long (>60sec) and dogs that discarded the sachet were more likely not vaccinated. Dogs chewing long rarely swallowed the sachet (12.4%), meanwhile most dogs that chewed very short swallowed the sachet (74.2%).

In the frame of this field trial with more than 1,100 baits handled, no adverse events were observed in dogs and vaccine exposures to humans that would require intervention did not occur. This adds to the high safety profile of this live vaccine when using the hand-out-and retrieve model [67]. Spillage of vaccine is not considered a source of contamination for potential contact to humans since the enveloped virus has a reduced viability in the environment. In the study area, the sandy floor, the high temperatures and the constant sunlight are further factors that decrease virus’ persistence. We did not detect rabies cases in vaccinated areas in three months after vaccination.

There are some limitations to this study. For statistical reasons, datasets with vaccination status “unknown” had to be removed thus leading to higher proportions of dogs being interested, consuming the bait and being assessed as vaccinated than if they were included.

**Fig 7:**
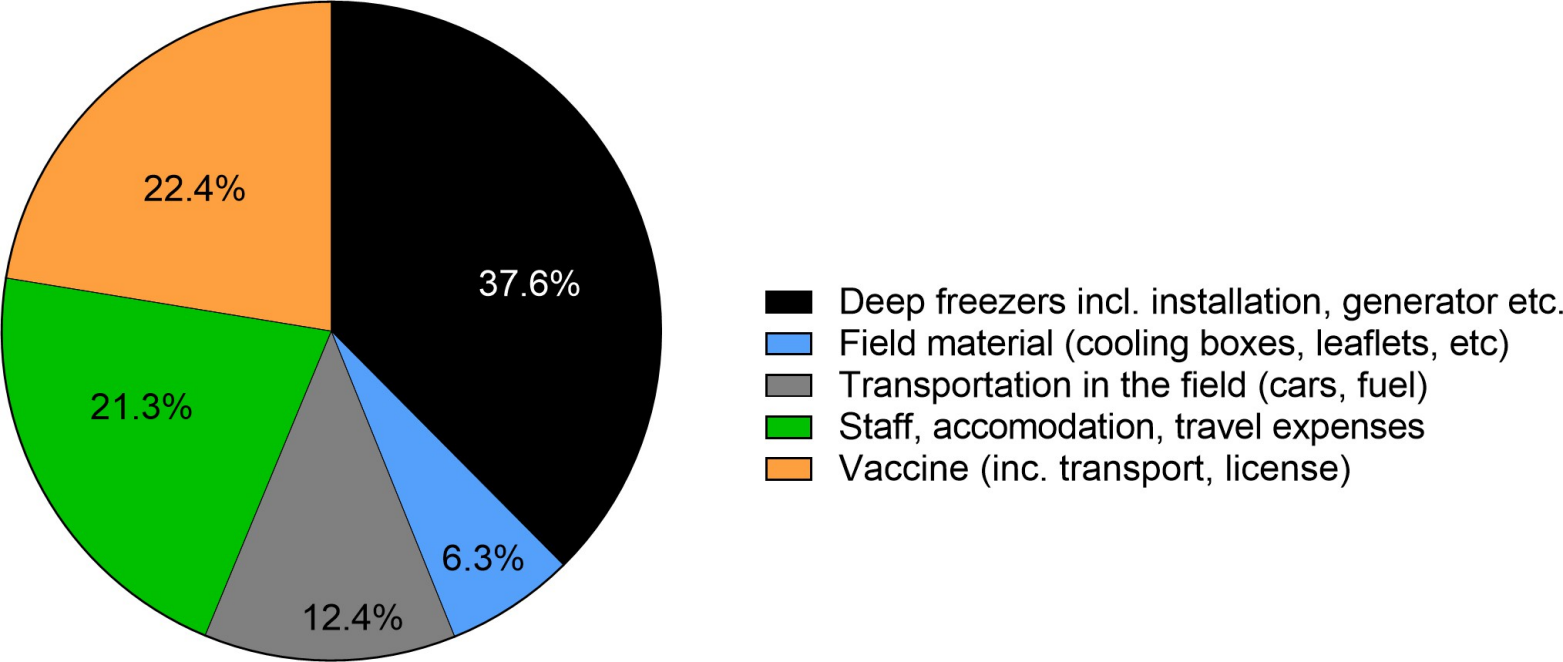
Pie-chart showing the percent shares of different costs, with a total budget of 51.045 US$.

Because of the research character of this field trial, an assessment of the costs and efficiency of ORV as a tool under the Namibian settings cannot be made. For example, deep freezers for the storage of vaccines constituted more than one third of the entire budget for this project (Fig 7) but are one-time investments that may not be required in other settings, depending on the prevailing logistical capacities and infrastructure. Also, due to the research component, more staff was involved than what would be needed if ORV was routinely used. In addition, accommodation and daily allowances were also provided to vaccinators; costs that may not be needed if regular staff is employed. As regards the costs for the oral vaccine baits, it is expected that prices could be reduced toward the minimum efficient scale if the market demand corresponds to the production capacities.

This research component with a required set of parameters to be typed into the mobile-phone app also prevented from vaccinating dogs in a shorter time interval when several dogs were presented for vaccination. Also, we did not attempt to vaccinate a certain proportion of the dog population and all values given for vaccination rates refer to those dogs that were offered a bait, but not to the entire dog population.

One aspect that was identified to limit the potential of ORV in the field was the requirement of owners’ consent prior to vaccination as was laid down in the study plan. Future campaigns should address this by indicating a general consent when the dog is free roaming at the time of vaccination. Another practical issue that emerged during the campaign was the provision of a vaccination certificate. Principally, the ORV method aims at the herd immunity and not the immune response in any individual dog, but specific ORV certificates may be issued during campaigns when ORV is included. In this field trial, both central-point vaccination as well as a door-to-door was used. As for the latter, with a highly dispersed human and dog population, partly absent dog owners, and distances between one and ten kilometers between individually vaccinated dogs (Fig 2C) if not even higher in other areas, this approach would be very inefficient and against the background of increasing costs for fuel, inappropriate under many settings. Rather, dog owners should be instructed to bring their dogs to a central point where parenteral and oral vaccination is conducted with a higher efficiency than parenteral alone. While dogs may be stressed due to the unfamiliar territory, other dogs and the transportation by leash as experienced before [44], in our study, we did not see a reduced bait uptake or vaccination rate when more dogs were present. However, to prevent negative influence dog owners could be instructed to keep their dogs at a certain distance.

In any case, a central point approach would again disregard those dogs that cannot be handled and brought to a vaccination point. To overcome this dilemma, the oral rabies baits could be handed over to the dog owners and vaccination would occur at their own premises, as has been demonstrated with non-vaccine baits in Tunisia [68]. A similar approach was also suggested for classical swine fever vaccinations to facilitate on-farm delivery in backyard pigs in remote areas [69]. For rabies, because of safety concerns such approach can only be envisaged for vaccines with a very high safety profile, so that a risk for humans is negligible [56]. While the vaccination success could not be controlled, this would still increase the herd immunity, particularly in the free-roaming hard-to-reach dogs. If dogs that act as super-spreaders are among those animals [70], targeting these highly connected dogs in the transmission networks would make vaccination campaigns more effective than random vaccination [71].

## 5. Conclusions

Even though planning and implementation of such a field trial in the midst of the COVID-19 pandemic represented a challenge, this pilot field trial of ORV in dogs in Namibia was very successful in terms of acceptance of the method, acceptability of the baits by dogs and the percentage of dogs offered a bait that were considered vaccinated. These results further support the strategic integration of ORV into dog rabies control programmes. Given the acceptance of the egg-flavored bait under various setting worldwide, ORV of dogs could become a game-changer in many African countries, where control strategies using parenteral vaccination alone failed to reach such vaccination coverage in the dog population that transmission was reduced and eventually controlled or eliminated, e.g. in West Africa [72], and Tanzania [15]. It is of note, that any study planning has to consider the availability of critical infrastructure to allow ORV baiting before the programme can be implemented.

Together with the recently published data on the epidemiology of rabies in Namibia [55], field data from dog vaccination campaigns [53,54], and immunogenicity of ORV in Namibian dogs [44] this study demonstrates Namibia’s efforts in piloting and executing applied rabies research. Future research on best-practice examples should entail the parallel application of ORV (for inaccessible dogs) and parenteral vaccination at central vaccination points during i) mass dog vaccinations and ii) cattle vaccinations at crush pens. Additionally, the effectiveness of an optimized ORV-only approach with owner consent and limited data acquis needs to be assessed. Such research including cost-benefit analyses will provide evidence whether and how to integrate ORV into Namibia’s rabies control programme.

## Supporting information

S1 Table The mean, minimum and maximum vaccination success rate (%) and the results of the univariate analysis of the selected independent variables (n = number of settings). (PDF)

S2 Table Odds ratios of the Multiple Logistic Regression (MLR) Model. (PDF)

S3 Table The effect of bait handling on vaccination success. (PDF)

## References

[1] Fahrion AS, Taylor LH, Torres G, Müller T, Durr S, Knopf L, et al. The Road to Dog Rabies Control and Elimination-What Keeps Us from Moving Faster? Front Public Health. 2017; 5: 103. doi: 10.3389/fpubh.2017.00103 28555183 PMC5430047

[2] Müller T, Freuling CM. 2020; Rabies in terrestrial animals. In: Fooks AR, Jackson AC, editors. Rabies—Scientific Basis of the Disease and its Management: Elsevier Academic Press. pp. 195–230.

[3] Rupprecht CE, Barrett J, Briggs D, Cliquet F, Fooks AR, Lumlertdacha B, et al. Can rabies be eradicated? Dev Biol (Basel). 2008; 131: 95–121. 18634470

[4] Fooks AR, Banyard AC, Horton DL, Johnson N, McElhinney LM, Jackson AC. Current status of rabies and prospects for elimination. Lancet. 2014; 384 (9951): 1389–1399. doi: 10.1016/S0140-6736(13)62707-5 24828901 PMC7159301

[5] World Health Organization. WHO Expert Consultation on Rabies. Third report. WHO Tech Rep Ser. 2018; 1012: 195.

[6] Morters MK, Restif O, Hampson K, Cleaveland S, Wood JL, Conlan AJ. Evidence-based control of canine rabies. a critical review of population density reduction. J Anim Ecol. 2013; 82 (1): 6–14. doi: 10.1111/j.1365-2656.2012.02033.x 23004351 PMC3579231

[7] Velasco-Villa A, Mauldin MR, Shi M, Escobar LE, Gallardo-Romero NF, Damon I, et al. The history of rabies in the Western Hemisphere. Antiviral Res. 2017; 146: 221–232. doi: 10.1016/j.antiviral.2017.03.013 28365457 PMC5620125

[8] Müller FT, Freuling CM. Rabies control in Europe: an overview of past, current and future strategies. Rev Sci Tech. 2018; 37 (2): 409–419. doi: 10.20506/rst.37.2.2811 30747138

[9] Freire de Carvalho M, Vigilato MAN, Pompei JA, Rocha F, Vokaty A, Molina-Flores B, et al. Rabies in the Americas: 1998–2014. PLoS Neglect Trop Dis. 2018; 12 (3): e0006271. doi: 10.1371/journal.pntd.0006271 29558465 PMC5877887

[10] Vigilato MAN, Clavijo A, Knobl T, Silva HMT, Cosivi O, Schneider MC, et al. Progress towards eliminating canine rabies: policies and perspectives from Latin America and the Caribbean. policies and perspectives from Latin America and the Caribbean. Philos Trans R Soc Lond B Biol Sci. 2013; 368 (1623): 20120143. doi: 10.1098/rstb.2012.0143 23798691 PMC3720041

[11] World Health Organisation. Mexico is free from human rabies transmitted by dogs. 2019;. Available: https://www.who.int/news/item/21-12-2019-mexico-is-free-from-human-rabies-transmitted-by-dogs. Accessed 12 November 2021.

[12] Reyes O. World Rabies Day: Latin America and the Caribbean on the cusp of eliminating rabies deaths. 2019;. Available: https://www3.paho.org/hq/index.php?option=com_content&view=article&id=15452:world-rabies-day-latin-america-and-the-caribbean-on-the-cusp-of-eliminating-rabies-deaths&Itemid=1926&lang=en. Accessed 12 November 2021.

[13] Hampson K, Coudeville L, Lembo T, Sambo M, Kieffer A, Attlan M, et al. Estimating the global burden of endemic canine rabies. PLoS Neglect Trop Dis. 2015; 9 (4): e0003709. doi: 10.1371/journal.pntd.0003709 25881058 PMC4400070

[14] Lembo T, Hampson K, Kaare MT, Ernest E, Knobel D, Kazwala RR, et al. The feasibility of canine rabies elimination in Africa. dispelling doubts with data. PLoS Neglect Trop Dis. 2010; 4 (2): e626. doi: 10.1371/journal.pntd.0000626 20186330 PMC2826407

[15] Sambo M, Ferguson EA, Abela-Ridder B, Changalucha J, Cleaveland S, Lushasi K, et al. Scaling-up the delivery of dog vaccination campaigns against rabies in Tanzania. PLoS Neglect Trop Dis. 2022; 16 (2): e0010124. doi: 10.1371/journal.pntd.0010124 35143490 PMC8865671

[16] Wallace RM, Cliquet F, Fehlner-Gardiner C, Fooks AR, Sabeta CT, Setién AA, et al. Role of Oral Rabies Vaccines in the Elimination of Dog-Mediated Human Rabies Deaths. Emerg Infect Dis. 2020; 26 (12): e1–e9. doi: 10.3201/eid2612.201266 33219786 PMC7706920

[17] Cliquet F, Guiot AL, Aubert M, Robardet E, Rupprecht CE, Meslin FX. Oral vaccination of dogs. a well-studied and undervalued tool for achieving human and dog rabies elimination. Vet Res. 2018; 49 (1): 61. doi: 10.1186/s13567-018-0554-6 30005701 PMC6045873

[18] Freuling CM, Hampson K, Selhorst T, Schroder R, Meslin FX, Mettenleiter TC, et al. The elimination of fox rabies from Europe. determinants of success and lessons for the future. Philos Trans R Soc Lond B Biol Sci. 2013; 368 (1623): 20120142. doi: 10.1098/rstb.2012.0142 23798690 PMC3720040

[19] Robardet E, Bosnjak D, Englund L, Demetriou P, Martín PR, Cliquet F. Zero Endemic Cases of Wildlife Rabies (Classical Rabies Virus, RABV) in the European Union by 2020: An Achievable Goal. Trop Med Infect Dis. 2019; 4 (4): 124. doi: 10.3390/tropicalmed4040124 31575054 PMC6958318

[20] MacInnes CD, Smith SM, Tinline RR, Ayers NR, Bachmann P, Ball DG, et al. Elimination of rabies from red foxes in eastern Ontario. J Wildl Dis. 2001; 37 (1): 119–132. doi: 10.7589/0090-3558-37.1.119 11272485

[21] Sidwa TJ, Wilson P, Moore GM, Oertli EH, Hicks BN, Rohde RE, et al. Evaluation of oral rabies vaccination programs for control of rabies epizootics in coyotes and gray foxes. 1995–2003. J Am Vet Med Assoc. 2005; 227 (5): 785–792. doi: 10.2460/javma.2005.227.785 16178403

[22] World Health Organization. Oral vaccination of dogs against rabies—Guidance for research on oral rabies vaccines and Field application of oral vaccination of dogs against rabies. 2007; Geneva, Switzerland.

[23] Baer GM, Brooks RC, Foggin CM. Oral vaccination of dogs fed canine adenovirus in baits. Am J Vet Res. 1989; 50 (6): 836–837. 2764336

[24] Rupprecht CE, Shaddock JS, Sanderlin DW, Hanlon CA, Niezgoda M, Schumacher CL. Oral rabies vaccination of dogs. Isr J Vet Med. 1998; 34: 228–239.

[25] Rupprecht CE, Hanlon CA, Blanton J, Manangan J, Morrill P, Murphy S, et al. Oral vaccination of dogs with recombinant rabies virus vaccines. Virus research. 2005; 111 (1): 101–105. doi: 10.1016/j.virusres.2005.03.017 15896409

[26] Aylan O, Vos A. Efficacy studies with SAD B19 in Turkish dogs. J Etlik Vet Microbiol. 1998; 9 (Special Issue Oral vaccination of dogs against rabies): 93–102.

[27] Faber M, Dietzschold B, Li J. Immunogenicity and Safety of Recombinant Rabies Viruses Used for Oral Vaccination of Stray Dogs and Wildlife. Zoonoses Public Health. 2009; 56 (6–7): 262–269. doi: 10.1111/j.1863-2378.2008.01215.x 19486317

[28] Yakobson BA, King R, Sheichat N, Eventov B, David D. Assessment of the efficacy of oral vaccination of livestock guardian dogs in the framework of oral rabies vaccination of wild canids in Israel. Dev Biol (Basel). 2008; 131: 151–156. 18634475

[29] Shuai L, Feng N, Wang X, Ge J, Wen Z, Chen W, et al. Genetically modified rabies virus ERA strain is safe and induces long-lasting protective immune response in dogs after oral vaccination. Antiviral Res. 2015; 121: 9–15. doi: 10.1016/j.antiviral.2015.06.011 26093157

[30] Frontini MG, Fishbein DB, Ramos JG, Collins EF, Torres JMB, Huerta GQ, et al. A Field Evaluation in Mexico of four Baits for Oral Rabies Vaccination of Dogs. Am J Trop Med Hyg. 1992; 47 (3): 310–316. doi: 10.4269/ajtmh.1992.47.310 1524144

[31] Matter HC, Kharmachi H, Haddad N, Ben Youssef S, Sghaier C, Ben Khelifa R, et al. Test of three bait types for oral immunization of dogs against rabies in Tunisia. Am J Trop Med Hyg. 1995; 52 (6): 489–495. doi: 10.4269/ajtmh.1995.52.489 7611552

[32] Matter HC, Schumacher CL, Kharmachi H, Hammami S, Tlatli A, Jemli J, et al. Field evaluation of two bait delivery systems for the oral immunization of dogs against rabies in Tunisia. Vaccine. 1998; 16 (7): 657–665. doi: 10.1016/s0264-410x(97)00259-4 9562683

[33] Gibson AD, Mazeri S, Yale G, Desai S, Naik V, Corfmat J, et al. Development of a Non-Meat-Based, Mass Producible and Effective Bait for Oral Vaccination of Dogs against Rabies in Goa State, India. Trop Med Infect Dis. 2019; 4 (3): 118. doi: 10.3390/tropicalmed4030118 31487795 PMC6789727

[34] Schuster P, Gülsen N, Neubert A, Vos A. Field trials evaluating bait uptake by an urban dog population in Turkey. J Etlik Vet Microbiol. 1998; 9 (Special Issue Oral vaccination of dogs against rabies): 73–81.

[35] Estrada R, Vos A, Leon RC de. Acceptability of local made baits for oral vaccination of dogs against rabies in the Philippines. BMC Infect Dis. 2001; 1 (1): 19. doi: 10.1186/1471-2334-1-19 11696244 PMC59655

[36] Knobel DL, Du Toit JT, Bingham J. Development of a bait and baiting system for delivery of oral rabies vaccine to free-ranging African wild dogs (Lycaon pictus). J Wildl Dis. 2002; 38 (2): 352–362. doi: 10.7589/0090-3558-38.2.352 12038135

[37] Bergman D, Bender S, Wenning K, Slate D, Rupprecht C, Heuser C, et al. Bait acceptability for delivery of oral rabies vaccine to free-ranging dogs on the Navajo and Hopi Nations. Dev Biol (Basel). 2008; 131: 145–150. 18634474

[38] Bender S, Bergman D, Vos A, Martin A, Chipman R. Field Studies Evaluating Bait Acceptance and Handling by Dogs in Navajo Nation, USA. Trop Med Infect Dis. 2017; 2 (2): 17. doi: 10.3390/tropicalmed2020017 30270876 PMC6082073

[39] Kasemsuwan S, Chanachai K, Pinyopummintr T, Leelalapongsathon K, Sujit K, Vos A. Field Studies Evaluating Bait Acceptance and Handling by Free-Roaming Dogs in Thailand. Vet Sci. 2018; 5 (2). doi: 10.3390/vetsci5020047 29734697 PMC6024691

[40] Haddad N, Khelifa RB, Matter H, Kharmachi H, Aubert MFA, Wandeler A, et al. Assay of Oral Vaccination of Dogs Against Rabies in Tunisia with the Vaccinal Strain SAD(_Bern)_. Vaccine. 1994; 12 (4): 307–309. doi: 10.1016/0264-410x(94)90093-0 8178551

[41] Hammami S, Schumacher C, Cliquet F, Tlatli A, Aubert A, Aubert M. Vaccination of Tunisian dogs with the lyophilised SAG2 oral rabies vaccine incorporated into the DBL2 dog bait. Vet Res. 1999; 30 (6): 607–613. 10596408

[42] Müller W, Güzel T, Auylan O, Kaya C, Cox J, Schneider L. The feasibility of oral vaccination of dogs in Turkey—a European Union supported project. J Etlik Vet Microbiol. 1998; 9 (Special Issue Oral vaccination of dogs against rabies): 61–73.

[43] Cliquet F, Gurbuxani JP, Pradhan HK, Pattnaik B, Patil SS, Regnault A, et al. The safety and efficacy of the oral rabies vaccine SAG2 in Indian stray dogs. Vaccine. 2007; 25 (17): 257–264. doi: 10.1016/j.vaccine.2006.12.054 17224221

[44] Molini U, Hassel R, Ortmann S, Vos A, Loschke M, Shilongo A, et al. Immunogenicity of the Oral Rabies Vaccine Strain SPBN GASGAS in Dogs Under Field Settings in Namibia. Front Vet Sci. 2021; 8: 737250. doi: 10.3389/fvets.2021.737250 34760958 PMC8573107

[45] Leelahapongsathon K, Kasemsuwan S, Pinyopummintr T, Boodde O, Phawaphutayanchai P, Aiyara N, et al. Humoral Immune Response of Thai Dogs after Oral Vaccination against Rabies with the SPBN GASGAS Vaccine Strain. Vaccines. 2020; 8 (4): 573. doi: 10.3390/vaccines8040573 33019605 PMC7711832

[46] Perera MA, Harischandra PA, Wimalaratne O, Damboragama SN. Feasibility of canine oral rabies vaccination in Sri Lanka—a preliminary report. Ceylon Med J. 2000; 45 (2): 61–64. 11051702

[47] Estrada R, Vos A, Leon R de, Mueller T. Field trial with oral vaccination of dogs against rabies in the Philippines. BMC Infect Dis. 2001; 1: 23. doi: 10.1186/1471-2334-1-23 11737869 PMC60992

[48] Smith TG, Millien M, Vos A, Fracciterne FA, Crowdis K, Chirodea C, et al. Evaluation of immune responses in dogs to oral rabies vaccine under field conditions. Vaccine. 2019; 37 (33): 4743–4749. doi: 10.1016/j.vaccine.2017.09.096 29054727 PMC5904025

[49] Darkaoui S, Boue F, Demerson JM, Fassi Fihri O, Yahia KI, Cliquet F. First trials of oral vaccination with rabies SAG2 dog baits in Morocco. Clin Exp Vaccine Res. 2014; 3 (2): 220–226. doi: 10.7774/cevr.2014.3.2.220 25003096 PMC4083075

[50] Chanachai K, Wongphruksasoong V, Vos A, Leelahapongsathon K, Tangwangvivat R, Sagarasaeranee O, et al. Feasibility and Effectiveness Studies with Oral Vaccination of Free-Roaming Dogs against Rabies in Thailand. Viruses. 2021; 13: 571. doi: 10.3390/v13040571 33805404 PMC8065424

[51] Abela-Ridder B, Balogh de K, Kessels JA, Dieuzy-Labaye I, Torres G. Global rabies control: the role of international organisations and the Global Strategic Plan to eliminate dog-mediated human rabies. Rev Sci Tech. 2018; 37 (2): 741–749. doi: 10.20506/rst.37.2.2837 30747112

[52] OIE. Chapter 2.1.17. Rabies (infection with rabies virus and other lyssaviruses) (NB: Version adopted in May 2018). Manual of Diagnostic Tests and Vaccines for Terrestrial Animals. 2018; Paris: OIE.

[53] Athingo R, Tenzin T, Shilongo A, Hikufe E, Shoombe KK, Khaiseb S, et al. Fighting Dog-Mediated Rabies in Namibia-Implementation of a Rabies Elimination Program in the Northern Communal Areas. Trop Med Infect Dis. 2020; 5 (1): 12. doi: 10.3390/tropicalmed5010012 31963400 PMC7157552

[54] Athingo R, Tenzin T, Coetzer A, Hikufe EH, Peter J, Hango L, et al. Application of the GARC Data Logger—a custom-developed data collection device—to capture and monitor mass dog vaccination campaigns in Namibia. PLoS Neglect Trop Dis. 2021; 14 (12): e0008948. doi: 10.1371/journal.pntd.0008948 33370285 PMC7793283

[55] Hikufe EH, Freuling CM, Athingo R, Shilongo A, Ndevaetela E-E, Helao M, et al. Ecology and epidemiology of rabies in humans, domestic animals and wildlife in Namibia, 2011–2017. PLoS Neglect Trop Dis. 2019; 13 (4): e0007355. doi: 10.1371/journal.pntd.0007355 30990805 PMC6486109

[56] Head JR, Vos A, Blanton J, Müller T, Chipman R, Pieracci EG, et al. Environmental distribution of certain modified live-virus vaccines with a high safety profile presents a low-risk, high-reward to control zoonotic diseases. Sci Rep. 2019; 9 (1): 6783. doi: 10.1038/s41598-019-42714-9 31043646 PMC6494895

[57] Namibia Statistics Agency. Namibia Inter-censal Demographic Survey 2016 Report. 2017;. Available: https://cms.my.na/assets/documents/NIDS_2016.pdf.

[58] Faber M, Pulmanausahakul R, Hodawadekar SS, Spitsin S, McGettigan JP, Schnell MJ, et al. Overexpression of the rabies virus glycoprotein results in enhancement of apoptosis and antiviral immune response. J Virol. 2002; 76 (7): 3374–3381. doi: 10.1128/jvi.76.7.3374-3381.2002 11884563 PMC136034

[59] Faber M, Faber ML, Papaneri A, Bette M, Weihe E, Dietzschold B, et al. A Single Amino Acid Change in Rabies Virus Glycoprotein Increases Virus Spread and Enhances Virus Pathogenicity. J Virol. 2005; 79 (22): 14141–14148. doi: 10.1128/JVI.79.22.14141-14148.2005 16254349 PMC1280225

[60] Ortmann S, Vos A, Kretzschmar A, Walther N, Kaiser C, Freuling C, et al. Safety studies with the oral rabies virus vaccine strain SPBN GASGAS in the small Indian mongoose (Herpestes auropunctatus). BMC Vet Res. 2018; 14 (1): 90. doi: 10.1186/s12917-018-1417-0 29534727 PMC5850969

[61] Gibson AD, Yale G, Vos A, Corfmat J, Airikkala-Otter I, King A, et al. Oral bait handout as a method to access roaming dogs for rabies vaccination in Goa, India: A proof of principle study. Vaccine X. 2019; 1: 100015. doi: 10.1016/j.jvacx.2019.100015 31384737 PMC6668228

[62] Bonwitt J, Bonaparte S, Blanton J, Gibson AD, Hoque M, Kennedy E, et al. Oral bait preferences and feasibility of oral rabies vaccination in Bangladeshi dogs. Vaccine. 2020; 38 (32): 5021–5026. doi: 10.1016/j.vaccine.2020.05.047 32513512

[63] Blancou J, Meslin FX. 1996;. Modified live-virus rabies vaccines for oral immunization of carnivores. In: Meslin FX, Kaplan MM, Koprowski H, editors. Laboratory techniques in rabies. Geneva, Switzerland: World Health Organization.

[64] Gibson AD, Mazeri S, Lohr F, Mayer D, Burdon Bailey JL, Wallace RM, et al. One million dog vaccinations recorded on mHealth innovation used to direct teams in numerous rabies control campaigns. PloS One. 2018; 13 (7): e0200942. doi: 10.1371/journal.pone.0200942 30048469 PMC6062050

[65] Bursac Z, Gauss CH, Williams DK, Hosmer DW. Purposeful selection of variables in logistic regression. Source Code Biol Med. 2008; 3: 17. doi: 10.1186/1751-0473-3-17 19087314 PMC2633005

[66] Solís Arce JS, Warren SS, Meriggi NF, Scacco A, McMurry N, Voors M, et al. COVID-19 vaccine acceptance and hesitancy in low- and middle-income countries. Nat Med. 2021; 27 (8): 1385–1394. doi: 10.1038/s41591-021-01454-y 34272499 PMC8363502

[67] Yale G, Lopes M, Isloor S, Head JR, Mazeri S, Gamble L, et al. Review of Oral Rabies Vaccination of Dogs and Its Application in India. Viruses. 2022; 14 (1): 155. doi: 10.3390/v14010155 35062358 PMC8777998

[68] Ben Youssef S, Matter HC, Schumacher CL, Kharmachi H, Jemli J, Mrabet L, et al. Field evaluation of a dog owner, participation-based, bait delivery system for the oral immunization of dogs against rabies in Tunisia. Am J Trop Med Hyg. 1998; 58 (6): 835–845. doi: 10.4269/ajtmh.1998.58.835 9660475

[69] Milicevic V, Dietze K, Plavsic B, Tikvicki M, Pinto J, Depner K. Oral vaccination of backyard pigs against classical swine fever. Vet Microbiol. 2013; 163 (1): 167–171. doi: 10.1016/j.vetmic.2012.12.005 23287388

[70] Brunker K, Hampson K, Horton DL, Biek R. Integrating the landscape epidemiology and genetics of RNA viruses. rabies in domestic dogs as a model. Proc R Soc B Biol Sci. 2012; 139 (14): 1899–1913. doi: 10.1017/S003118201200090X 22814380 PMC3526958

[71] Warembourg C, Fournié G, Abakar MF, Alvarez D, Berger-González M, Odoch T, et al. Predictors of free-roaming domestic dogs’ contact network centrality and their relevance for rabies control. Sci Rep. 2021; 11 (1): 12898. doi: 10.1038/s41598-021-92308-7 34145344 PMC8213792

[72] Mbilo C, Coetzer A, Bonfoh B, Angot A, Bebay C, Cassamá B, et al. Dog rabies control in West and Central Africa: A review. Acta Trop. 2020; 105459. doi: 10.1016/j.actatropica.2020.105459 32404295

