# Supplementary material for: Oral rabies vaccination of dogs—Experiences from a field trial in Namibia
Source: PLoS Negl Trop Dis. 2022 Aug 22;16(8):e0010422. doi: 10.1371/journal.pntd.0010422 (PMC9436088; doi:10.1371/journal.pntd.0010422)
Supplement: S1 Table — (PDF) [file pntd.0010422.s001.pdf]

Supplementary table 1. The mean, minimum and maximum vaccination success rate (%) and the results of the univariate analysis of the selected independent variables (n = number of settings)

Variable	n	mean	minimum	maximum	p-value (statistical test)
date	**8**	82.5	69.8	89.0	0.0048 (Chi²)
period of the day	**4**	82.5	81.0	84.5	0.8790 (Fisher)
team	**4**	82.5	80.2	85.3	0.4263 (Chi²)
level of supervision [1)]	**3**	82.5	73.3	82.8	0.4044 (Chi²)
social status [2)]	**2**	82.6	80.8	85.9	0.0494 (Fisher)
Size [3)]	**3**	82.4	77.2	87.2	0.0166 (Chi²)
Sex [4)]	**2**	82.9	82.0	84.5	0.3700 (Fisher)

1) 1 dog with no entry for level for supervision; not included in the statistical analysis'

2) 26 dogs with no entry for social status; not included in the statistical analysis

3) 14 dogs with no entry for size; not included in the statistical analysis

4) 30 dogs with no entry for sex; not included in the statistical analysis
