# Supplementary material for: Oral rabies vaccination of dogs—Experiences from a field trial in Namibia
Source: PLoS Negl Trop Dis. 2022 Aug 22;16(8):e0010422. doi: 10.1371/journal.pntd.0010422 (PMC9436088; doi:10.1371/journal.pntd.0010422)
Supplement: S2 Table — (PDF) [file pntd.0010422.s002.pdf]

Supplementary table 2: Odds ratios of the Multiple Logistic Regression (MLR) Model.

coefficient	Variable	Odds ratios	95% CI (profile likelihood)
β0	Intercept	0.4756	0.1819 to 1.110
β1	B[21.10.2021]	0.6802	0.2724 to 1.870
β2	B[22.10.2021]	0.7462	0.3063 to 2.016
β3	B[23.10.2021]	0.4022	0.1543 to 1.139
β4	B[25.10.2021]	0.3564	0.1257 to 1.065
β5	B[26.10.2021]	0.4483	0.1568 to 1.350
β6	B[27.10.2021]	0.5079	0.1893 to 1.467
β7	B[28.10.2021]	1.125	0.4302 to 3.206
**β8**	**C[multiple]**	**0.6188**	**0.4203 to 0.8984**
β9	D[large]	1.254	0.6827 to 2.215
**β10**	**D[small]**	**0.5862**	**0.3905 to 0.8656**
