# Supplementary material for: Oral rabies vaccination of dogs—Experiences from a field trial in Namibia
Source: PLoS Negl Trop Dis. 2022 Aug 22;16(8):e0010422. doi: 10.1371/journal.pntd.0010422 (PMC9436088; doi:10.1371/journal.pntd.0010422)
Supplement: S3 Table — (PDF) [file pntd.0010422.s003.pdf]

Supplementary table 3: The effect of bait handling (consumption - amount of bait matrix consumed; chewing time – total time [seconds] dog spend chewing on the bait; sachet – fate of sachet after bait consumption, discarded or swallowed) on vaccination success (%); N – number of dogs offered a bait, n – number of dogs considered successfully vaccinated. Data sets with an entry 'unknown' for these variables were excluded from statistical analysis.

variable	N	n	%	p-value
Consumption				
- complete (100%)	731	682	92.3	
- most (>50%)	133	119	89.5	0.2822 (Chi²)
- little (<50%)	10	9	90.0	
Chewing time				
- long (>60sec)	89	76	85.4	
- medium (30-60sec)	318	297	93.4	0.0039 (Chi²)
- short (10-30sec)	225	217	96.4	
- very short (<10sec)	242	219	90.5	
sachet				
- discarded	425	384	90.4	0.0135 (Fisher)
- swallowed	442	419	94.8	
