# Supplementary material for: Oral rabies vaccination of dogs—Experiences from a field trial in Namibia
Source: PLoS Negl Trop Dis. 2022 Aug 22;16(8):e0010422. doi: 10.1371/journal.pntd.0010422 (PMC9436088; doi:10.1371/journal.pntd.0010422)
Supplement: Attachment — Submitted filename: tw2022-_rebuttal.pdf [file pntd.0010422.s004.pdf]

Friedrich-Loeffler-Institut | Postfach 1318 | 17466 Greifswald-Insel Riems

PlOS NTD Editorial Board

Institut für molekulare Virologie und Zellbiologie
Institute of Molecular Virology and Cell Biology
Leiter: Prof. Dr. Stefan Finke

Referat od. Labor: Nationales Referenzlabor für Tollwut
bearbeitet von: Dr. Conrad Freuling
Telefon: 038351 7 1659
Fax: 038351 7 1174
E-Mail: Conrad.Freuling@fli.de
Zeichen od. AZ: tw2022-8_cover_letter
Datum: 13-06-2022

Submission of a revised version of PNTD-S-22-00642

Dear editors,

Thank you for giving us the opportunity to submit a revised version of the manuscript according to the reviewers' comments. We clarified that we did not

All authors have seen and approved the submitted version.

I look forward hearing from you.

Yours sincerely

Conrad Freuling

Hauptsitz - Insel Riems
Südufer 10
17493 Greifswald - Insel Riems
Telefon: +49 38351 7-0
Fax: +49 38351 7-1151
www.fli.bund.de

Standort Braunschweig
Bundesallee 50
38116 Braunschweig
Telefon: +49 531 596-3102
Fax: +49 531 596-3299
www.fli.bund.de

Standort Celle
Dörnbergstraße 25/27
29223 Celle
Telefon: +49 5141 3846-0
Fax: +49 5141 3846-117
www.fli.bund.de

Standort Jena
Naumburger Straße 96a
07743 Jena
Telefon: +49 3641 804-2100
Fax: +49 3641 804-228
www.fli.bund.de

Standort Mariensee
Höltystraße 10
31535 Neustadt
Telefon: +49 5034 871-0
Fax: +49 5034 871-143
www.fli.bund.de

Rebuttal

Reviewer #1: Line 122: How were rural and suburban defined?
A: The northern communal areas in Namibia are predominately rural comprising of homesteads where larger families live. Only few places are assigned as towns and can be regarded as urban, and the dense population around the centres were considered as semi-urban.
Lines 122 – 125: Please provide more information about these communities. What is human population? Estimated dog population? Area? Any significant cultural considerations?
A: We have added information on the structure of these regions. A more detailed study on KAP towards rabies and dog population will be published elsewhere.
Figure 1: Please confirm if these maps are open-access. Some google map baselayers cannot be published without written approval.
A: The baselayer is open street map and no further approval is needed.
Figure 1: The colors do not seem to provide any information relevant to the study results. If this is true, suggest to select just one color. If not true, please explain in a footnote and methods section.
Figure 1: The sites are very difficult to view given how small and dispersed they are. Suggest to zoom in either by cropping the maps differently or having multiple inset images of these communities.
A: Figure 1 was revised accordingly.
Lines 151 – 153: was there evidence that egg-alone resulted in sub-par uptake? This is unfortunate, as it is one more logistical constraint for countries considering replicating this approach.
A: The general acceptance of the “egg-only bait” was very similar to the “flavoured” bait. The latter seemed to increase the initial awareness to the bait.
Line 164: please add additional information about what assay was performed to validate the titer of the vaccines.
A. A reference was added.
Line 175: 4b4? Define
A: Changed to four-wheel drive.
Section 2.4: what training did the vaccinators receive?
A: this was specified in section 2.5. We have moved the section up.
What preventive measures did they receive in regards to working with a modified live vaccine? Were vaccination team members required to be vaccinated against rabies?
A: No, that was not a requirement, because there was no direct interaction with dogs anticipated. Also, based on the safety profile of the vaccine, and precautionary measures e.g. wearing gloves, a vaccination was not considered as required. Still, the vast majority of team members were vaccinated.
Some of this information is found in section 2.5, but it seems more appropriate in section 2.4
A: Changed as suggested.
Line 180 – 181: I am assuming these DD and CP methods used parenteral vaccines? Please clarify
A: In this study, for logistical reasons (e.g. Covid-19) we only assessed the oral vaccine acceptance. This is stated in line 205. Further studies will look at combining both oral and parenteral vaccinations.
Line 195: what is the recommendations if a bait is still unused after the second day? Refrigerate and re-use, or dispose?
A: In our study settings, the bait was only used during the individual vaccination lasting no longer than minutes to a few hours. Baits not accepted were retrieved, cleaned with water and offered again to another dog. Depending on the climatic conditions, the vaccine virus titre may remain above the minimum effective dose for up to 4-6 days, but under African conditions, one would rather dispose. Baits left-over at the end of the day, were kept at refrigerator

temperatures overnight and used immediately the next day. Hence, no baits were used more than 36 hours after thawing up.
Line 196 – 197: this statement is inconsistent with the described methods. If owners brought dogs to a central location, why were they given oral vaccines instead of parenteral vaccines?

A: Please, see comment above. After announcement of the vaccination study people gathered at central places and dogs were either brought or followed their owners, and here we assessed the acceptance of the oral vaccination only. It was not a regular vaccination campaign.
Line 200: Per WHO and OIE recommendations, were these owners and community members also educated on what to do if an exposure were to occur, either through their recently-vaccinated-dog or a found-bait in the community?

A: Yes, informing the community about the scope of the study and any safety issues was the core of communication through verbal information and a leaflet in both the local language Oshiwambo and English.
Line 248: this is a very high p-value for inclusion in a final MLR model. Is there a citation for this approach, or at least commentary on why such a high inclusion cutoff was used?

A: It is standard practise to use a cutoff value of 0.2 or 0.25 for selecting the variables during the univariate analysis. If the standard 0.05 cutoff is used, it may be possible that a variable individually insignificant but in multivariable setup is significant or vice versa.

Reviewer #2: The methods are generally clearly articulated but I have a few specific comments (below). The study design is appropriate to address objectives around the acceptability of oral vaccination (both to dogs and owners) and its potential value in scaling up mass dog vaccination in rural communities.

It would be good to include in the abstract a concise description of how bait update and vaccination were assessed (e.g. direct observation) to clarify that no post-vaccination serological data were included in this analysis (which is something some readers may be expecting to see).

A: direct observation was included in the abstract.
Line 155, section 2.3. Provide a reference for methods used for checking quality of baits and vaccine titre.

A: A reference was included.
Fig. 1: More information needs to be provided in the legend with larger labels on the map to indicate locations, including the different community names. Given that >1,000 dogs were vaccinated, it's not clear how the circles explicitly relate to individual dogs – presumably there are multiple circles that overlap? Some further information in the legend would be helpful.

A: The map was changed accordingly.
Section 2.5 Vaccinations. It would be useful to provide a description as to what type of central point locations were selected. Were these locations where you might expect to find 'ownerless' dogs?

A: Please, see comment above. Central places were selected based on previous mass dog vaccination campaigns and mostly comprised of village centres or chrush pens (places where cattle are regularly vaccinated against FMD and other livestock diseases).
How soon after vaccination were the discarded sachets retrieved?

A: The retrieval was done immediately after the sachets were dropped.
Section 2.7 Evaluations and statistical analysis. Include how long (approximately) the animal was observed if it did not take the bait immediately .
For example, if it walked away, was the bait retrieved immediately, or was the dog/bait observed for a specified period of time to see if the dog came back to it.

A: The observation was usually done after 1-2 minutes but was stopped after three minutes.

Include some description of how free-roaming and ownerless dogs were identified and classified. Both 'free-roaming' and 'ownerless' seem quite difficult classifications to make if the evaluation was made while administering baits door-to-door or at a central point.
A: The vast majority (>90%) of dogs in Namibia have owners but are free roaming, and this is based on a recent KAP study that will be published. The challenge are dogs that cannot be restraint for vaccination. During D2D, we could see whether dogs were confined or free roaming. Only at central points, there were a few dogs without owners and these were identified as 'ownerless' by locals present.

Reviewer #1: Line 279: Is this the correct use of "datasets"? The sentence is confusing.

A: we do not really see a need to revise.

Figure 2, panel (a) – showing this data on the log scale does allow for the full range of data to be presented, but its difficult for the reader to tell the median distance for each day due to presentation of data on the log scale. Suggest to find a way to present the median values, which is of more importance than the total (presented in the box). What is the reason to explore distance between dogs by day? Was there an a prior assumption that there would be a significant relationship? I do not understand why this association was explored and how it relates to the act of vaccinating dogs by oral route.

A: We agree with the reviewer's last statement that distance alone is in no relation to the route of vaccination. Also, we did not assume or test for any significance. However, we wanted to use these data to inform readers on the logistical challenges that need to be overcome when vaccinating dogs. In these hard-to-reach communities an owner-based oral vaccination may be a realistic and cost saving alternative. As suggested, we have modified the figure and displayed the mean instead of the total distance.

Line 287: it is unclear. Were these confined dogs also given ORV? If so, why, as it seems like they could have been vaccinated through the parenteral route.

A: See comments before. Most confined dogs could have been given the vaccine parenterally, but based on the study design, we wanted to assess the uptake and vaccination by the oral route. On the other hand, confinement per se is not a guarantee that the dog can be vaccinated parenterally, as the confinement may be the entire homestead which sometimes has a size of 1000m^2 and owners were not able to restrain the dogs.

Line 290: this idea of calling crush pens a "central point" vaccination may not be relatable to a majority of readership. It would be very interesting to have more explanation of this modified approach to central point, preferably detailed in the methods section under vaccination approaches. Otherwise, it appears like dogs were easily accessible to parenteral, but given ORV, which does not comply with the stated objectives.

See comments before. Because ORV could not be linked to parenteral mass dog vaccination campaigns we restricted the objective to the acceptance of this method for owners and dogs and to assess the vaccination in dogs offered a bait.

Line 291: that's a long distance! I wonder if baiting by motorbike would improve efficiency.

A: Principally, yes, a motorbike would be a lot cheaper than a four-wheel drive truck. However, the road network with very sandy pits during the dry season and flooding during the rainy season represent challenges for motorbikes and they are hardly used in this part of Africa.

Line 299: "unkNown"

A: Changed!

Line 296 – 299: What was the estimated vaccination coverage among the inaccessible, free-roaming dog population?

A: We did not stratify the dogs into this category.

Line 303: were unperforated blisters re-used? Were these 45 vaccines reused?

A: Untouched baits were used again, but not partly consumed baits nor unperforated blisters.

Line 306 and 307: what were the a prior assumptions as to why time of day and day of campaign would be related to ORV acceptance? These seem like unrelated and random associations. Its not clear why they were explored and, had they been significant, likely would have represented spurious results. I strongly suggest to provide an explanation for some of these analyses in the methods, otherwise it appears like data fishing.

A: Time of day was assessed as a parameter because both the feeding of dogs (if dogs are fed in the evening, they may be more interested in baits in the morning etc.) and their circadian activity level (e.g. reflecting the daily temperature almost reaching 40°C at ca. 2:00 pm) may influence bait interest and uptake. So the question would be as to optimize an ORV campaign by using a specific time. Day of campaign was included as the teams had no previous experience in approaching and offering dogs a bait. Thus, with increasing time the teams would collect more experience and approached the dogs more efficiently (offering the bait at the right time before the animal runs away).

Figure 3: Why does this figure use confidence intervals, while other figures with similarly presented data do not? The presentation of the data in the figures is inconsistent. That is not necessarily a problem, but seems unnecessary.

A: We are not clear what the reviewer means. Confidence intervals were provided for all analyses on bait interest, consumption and the vaccination assessment

Line 327: was "chewing very long" associated with size or age? It seems odd that chewing a long time would REDUCE the chance that a sachet was punctured.

A: No, there was no association. Those dogs seem to be focussed on carefully eating the bait matrix and separating it from the blister.

Line 328: "more likely not vaccinated" is confusing. Why not say, "less likely to be considered vaccinated"

A: Changed.

Figure 5: Please clarify in the y-axis that this is the vaccination percent in the dog population. As presented now, it appears to be the percent of dogs, which cannot be more than 100% in each category.

A: Changed as suggested.

Results – what were the results of the sequencing from rabid dogs after the campaign (mentioned in methods?

A: There were three rabid cases in 3 month post vaccination in these regions Omusati and Oshana. All three were identified as local dog rabies strains.

How many community vaccine contact events were reported?

A: None reported.

How many adverse events to vaccine were reported?

A: There were no adverse events reported.

Reviewer #2: It would be good to include a breakdown of the number of dogs vaccinated door-to-door and those at central-point locations.

A: Unfortunately, this was not assessed in the data recording

Line 327 and Fig 5: Consistency needed in terms of classification of the time baits were chewed e.g. the text refers to 'very long' (> 60 secs) but results are only provide for 'long' in Fig 5. Similarly, in the supplementary information 'long' refers to > 60 secs.

A: Changed for consistency.

I could not find reference to results in relation to rabies diagnosis and virus characterisation either in the main manuscript of supplementary tables. If no samples were obtained, I would suggest removing reference to this in the methods.

A: … no reply? …

Reviewer #1: Line 352: is this a fair comparison? Your egg baits were dipped in an additional meat-based sauce. Unfortunately, I believe this makes your comparison to the other studies inappropriate. It is, however, appropriate to note that ORV baits in numerous studies, including this one, have shown a high acceptance rate; particularly when they are tailored to local dog preferences.

A: We have toned down the comparative statement.

Line 355: this statement is misleading. You had a vaccination coverage of 73% among the dogs that were offered a bait. This is not the same as a vaccination coverage in the community. The study does not appear to have assessed community vaccination coverages achieved by this campaign. How many dogs are in the community? Is there a census? Was a post-vaccination survey conducted to determine the vaccination coverage?

A: The reviewer states that our statements are misleading. We have therefore added "dogs that were offered a bait" throughout the manuscript. However, we did not state anywhere in the manuscript a "vaccination coverage". For clarity to the reviewer, the initial study plan was indeed a combination of parenteral MDV (incl. dog marking) plus post vaccination monitoring and ORV. For various reasons this could not be performed and we changed to a baiting assessment study only.

There is no census of dogs at the community level nor have we assessed community vaccination coverage. Preliminary results from a KAP study suggests a dog:human ration of 1:4.4 which will be used in upcoming studies.

Line 361: it is odd that body condition score was not collected during this study. Other papers have speculated that lower BCS may relate to better uptake due to hunger and reliance on scavenging for food resources. Why was BCS not collected? And could this be a possible explanation for the difference in uptake from Thailand?

A: Yes, BCS would have been another parameter to assess, but was not applied here, because this appeared more time consuming than a simple size classification.

Line 369: Why was uptake by peri-urban vs rural not explored in this analysis? The results indicate that it was part of the data collection process, but none of these results are presented. The discussion indicates that success was lower in peri-urban areas, but there is no data presented to share this. This would be a significant finding, much more so than vaccinations by day or team or hour. Strongly suggest that this is better explored in the analysis and discussed.

A: The data collection did not comprise of peri-urban vs rural. Generally, we visited rural areas and only a few teams visited the peri-urban areas on a single day. Hence, the available data is too limited for detailed analysis and comparison

Lines 371 – 380: the authors should clarify what is mean by small, medium and large. The methods indicate this was weight-based (kg). Could "small" dogs not just be thinner and therefore more aggressive to receive food? Minor clarification/explanation in the methods and here would be helpful to the reader.

A: This was added to the methods.

Lines 377 – 380: these sentences are just repeating the results and offer no insight into why this is relevant to the study or future oral vaccination campaigns. Personally, I don't see the relevance at all, but if the authors think this is a noteworthy finding, it is not apparent as-written. I suggest to drop this analysis, or better explain why this is anything more than a spurious association.

A: …. Reply? …

Line 390: the lack of considerations for costs and logistics make any claims about the feasibility of ORV in Namibia moot. Plenty of approaches can be enacted to improve vaccination coverages, but we do not apply them because they are too costly or logistically challenging. There is plenty of great data in this paper to suggest further exploration of the role of ORV in Namibia's dog vaccination program. But this study should avoid any suggestion that the data shows that ORV in Namibia should be incorporated into the current strategy. Without cost-

effectiveness considerations and consideration for the availability of critical infrastructure to allow ORV baiting, this study cannot claim the approach should be implemented.

A: We agree with the reviewer's concerns and have modified the wording accordingly.

Line 431: this statement is misleading. The vaccination coverage of dogs in this community was not assessed. The authors have assessed the vaccine uptake among dogs presented to them. This should be clarified throughout. No effort appears to have been undertaken to estimate the community vaccination coverage after this campaign.

A: We changed the wording accordingly. As the objective of the study was not to vaccinate a number of dogs or to fully vaccinate a certain region, we did not assess vaccination coverage post vaccination.

Lines 432 – 436: statements such as these would be MUCH more impactful if a simple cost component were added to this paper. In the absence of considering cost, the interpretation of impact is severely limited.

A: Again, we do not disagree with the reviewer that costs are a critical component. But these studies hopefully will be conducted in Namibia and elsewhere. However, the precondition is that the vaccine is acceptable and this is the case.

Reviewer #2: The study was carried out as a campaign that only involved oral vaccination, which has generated some valuable data, but it does have important implications in terms of interpretation and generalisability. The discussion includes consideration of some of the limitations of the study design, but a key point that has not been fully discussed is that many of the dogs reached through the oral vaccination approach may have been accessible through parenteral vaccination, particularly after the community sensitisation and advertising activities that were conducted. So, while the study clearly demonstrates acceptance of dogs and owners and the potential value of this type of approach, the discussion does not address explicitly whether or how the data provides insight on the coverage that might be expected to be achieved in 'hard-to-reach' rural dog populations or the 'supplementary' value of oral vaccination over and above parenteral vaccination.

A: Please see comments before.

Editorial and Data Presentation Modifications?</br>

Line 96. Shift 'also' to mid part of the sentence e.g. 'Attractiveness and update ….have also been tested'. I would perhaps also add a phrase at the end of the sentence to indicate where/in what settings these have been tested.

A: Changed as suggested.

Line 99: 'Studies' should be 'study'

A: Changed

Line 108: 'where THE percentage'

A: Changed

Line 173: Remove 'of' following 'comprised'

A: Changed

Line 329: Include 'chewed FOR A very short time…'

A: Changed

Reviewer #1: OVERALL: This paper describes the continued application of research findings to improve dog vaccination coverage in Namibia. The evolution of the Namibian rabies control program is exciting and provides an example for success in the region. The logistical efforts undertaken to conduct this study are much-appreciated and the results provide more evidence to the literature that ORV is a safe and effective method of vaccinating free-roaming dogs. While the paper is easy to read and is a very important topic, I have several concerns with the analysis and presentation of the data. These are detailed below, but summarized here as well:

1) Several components of the analysis seem to be rather meaningless and the authors do not adequately describe why they had any a priori interest in these assumptions. As expected, the results were not significant. If the associations have no plausible reason to be related, and the analysis shows they are not related… what is the point? This applies specifically to analyses related to time of day, day of campaign, distance by day, and chewing time. These topics, specifically, should be much better explained and rationalized if the authors feel they are important to remain in the study.

A: See detailed comments before.

2) Without consideration for cost, the authors are severely limited in statements suggesting that this is a feasible approach to dog vaccination. Even a simple table with costs incurred to operate this campaign would be incredibly useful for the rabies community and for understanding the logistical and cost barriers to expanding ORV use. Without a cost component, the authors will need to be very careful about over-interpreting the impact of these findings.

A: See detailed comments before.

3) The authors did not use egg baits. They used egg baits dipped in meat sauces. While interesting, this diminishes the comparability to other studies. Use caution when making such comparison statements in the article.

A: The egg-bait was further “adapted” to local preferences. This was added to the discussion.

4) the authors did not assess the post-campaign vaccination coverage in this community. Many statements could easily be taken out of context to imply that this campaign achieved >70% vaccination coverage. The authors methods and analysis only imply that 70% of dogs offered a bait were vaccinated. If the authors are certain that they approached EVERY dog in the community, then this claim may be appropriate. Unless this can be clearly and confidently stated, then the coverage reported here is not equivalent to the community vaccination coverage.

A: We clarified our wording that we did not assess vaccination coverage in the population. This was not the objective of this study.

Reviewer #2: This is a well-written manuscript that presents important data on oral rabies vaccination of dogs, which is an area of growing interest in relation to scaling up of mass dog vaccination in order to reach international targets of zero human deaths from canine rabies by 2030 (“Zero by Thirty”. The study has been executed well and is suitable for publication. A few minor comments/suggestions are included in the sections above.

A: We are grateful for the positive feedback.
